# Dexmedetomidine inhibits the NF-κB pathway and NLRP3 inflammasome to attenuate papain-induced osteoarthritis in rats

**DOI:** 10.1080/13880209.2019.1651874

**Published:** 2019-09-23

**Authors:** Fang Cheng, Feng-Feng Yan, Yue-Peng Liu, Yan Cong, Ke-Fu Sun, Xue-Ming He

**Affiliations:** a Department of Pain Clinic, The Affiliated Lianyungang Oriental Hospital of Xuzhou Medical University, Lianyungang, China;; b Center for Clinical Research and Translational Medicine, The Affiliated Lianyungang Oriental Hospital of Xuzhou Medical University, Lianyungang, China;; c Department of Traditional Chinese and Western Medicine, The Affiliated Lianyungang Oriental Hospital of Xuzhou Medical University, Lianyungang, China;; d Department of Orthopedic, The Affiliated Lianyungang Oriental Hospital of Xuzhou Medical University, Lianyungang, China;; e Department of Geratology, The Affiliated Lianyungang Oriental Hospital of Xuzhou Medical University, Lianyungang, China

**Keywords:** Joint pain, inflammation, paw withdrawal mechanical threshold, paw withdrawal thermal latency, articular cartilage

## Abstract

**Context:** Dexmedetomidine (Dex) has been reported to have an anti-inflammatory effect. However, its role on osteoarthritis (OA) has not been explored.

**Objective:** This study investigates the effect of Dex on OA rat model induced by papain.

**Materials and methods:** The OA Wistar rat model was induced by intraluminal injection of 20 mL of papain mixed solution (4% papain 0.2 mL mixed with 0.03 mol L^−1^ l-cysteine 0.1 mL) into the right knee joint. Two weeks after papain injection, OA rats were treated by intra-articular injection of Dex (5, 10, or 20 μg kg^−1^) into the right knee (once a day, continuously for 4 weeks). Articular cartilage tissue was obtained after Dex treatment was completed.

**Results:** The gait behavior scores (2.83 ± 0.49), PWMT (15.2 ± 1.78) and PTWL (14.81 ± 0.92) in H-DEX group were higher than that of OA group, while Mankin score (5.5 ± 0.81) was decreased (*p* < 0.05). Compared with the OA group, the IL-1β (153.11 ± 16.05 pg mg^−1^), IL-18 (3.71 ± 0.7 pg mg^−1^), IL-6 (14.15 ± 1.94 pg/mg) and TNF-α (40.45 ± 10.28 pg mg^−1^) levels in H-DEX group were decreased (*p* < 0.05). MMP-13, NLRP3, and caspase-1 p10 expression in Dex groups were significantly lower than that of OA group (*p* < 0.05), while collagen II was increased (*p* < 0.05). p65 in the nucleus of Dex groups was significantly down-regulated than that of OA group (*p* < 0.05).

**Discussion and Conclusions:** Dex can improve pain symptoms and cartilage tissue damage of OA rats, which may be related to its inhibition of the activation of NF-κB and NLRP3 inflammasome.

## Introduction

Osteoarthritis (OA) is a common joint disease that occurs in the elderly. About 15% of the world's population suffers from OA (Lawrence et al. 2008). Although OA is the most common form of disability in the elderly, no drug exists to slow the progression, or reverse the OA disease process (McCarthy and Cheung 2009). Once the development of OA enters the end stage, joint replacement is the only effective treatment. At present, non-surgical treatment of OA is mostly aimed at alleviating pain caused by inflammatory reaction and alleviating symptoms. Exploring safe and effective therapeutic drugs has become an urgent problem to be solved in the early treatment of OA.

The main pathological feature of OA is the degeneration of articular cartilage, and the synovial membrane and subchondral bone are simultaneously affected (Buckwalter and Mankin 1998). Current research suggests that OA is a chronic inflammatory disease (Benito et al. 2005). A variety of inflammatory factors play an important role in the pathogenesis of OA. Interleukin-1 (IL-1) is considered to be the initiating factor of inflammation regulation that can be recognized by pattern recognition receptors (PRRs) to participate in the inflammatory effects of cascade amplification. IL-1β is a major member of the IL-1 family that has been shown to play an important role in the progression of OA (Margerie et al. 1997). A large amount of IL-1β can be detected in the joint fluid of patients with OA (Ramonda et al. 2014). The high expression of IL-1β was also found in the cartilage tissue of OA, and the concentration of IL-1β in the tissue directly reflected the severity of OA (Towle et al. 1997). IL-1β has been widely used in *in vitro* models to induce chondrocyte inflammatory response (Zhou et al. 2008).

Nod-like receptor pyrin domain 3 (NLRP3) inflammasome is considered to be a molecular switch that regulates the production of inflammation, and is a hot spot in the field of inflammatory immunity research in recent years. Activation of NLRP3 inflammasome leads to cleavage of caspase-1. Activated caspase-1 is capable of cleavage and processing of pro-IL-1β and IL-18 to promote their cleavage into mature IL-1β and IL-18 and secrete cells (Zhou et al. 2011). These cytokines are further involved in the regulation of the inflammatory cascade. NLRP3 has been shown to play an important role in the inflammatory response of various diseases including osteoarthritis. Based on this, Zhang et al. (2012) proposed that inhibition of NLRP3 inflammasome activity can improve renal dysfunction in diabetic nephropathy. Mridha et al. (2017) believed that inhibition of NLRP3 inflammasome activation can reverse liver inflammation and improve liver fibrosis. Sun et al. (2017) also found that inhibition of NLRP3 inflammasome activation can improve osteoarthritis disease progression in destabilization of the medial meniscus (DMM) model of osteoarthritis. Therefore, as the core of the inflammatory response, NLRP3 inflammasome may provide new targets for the treatment of OA (Bougault et al. 2012; McAllister et al. 2018).

Dexmedetomidine (Dex) is a highly selective α2 receptor agonist, and its research on anti-inflammatory and analgesic has been reported. Dex acts on the posterior synaptic and interneuronal nerves of the spinal cord without post-synaptic membrane α2 adrenergic receptors, which inhibits pain signaling to brain conduction and weakens intra-articular injection of complete Freund's adjuvant (CFA)-induced hyperalgesia in monoarthritis (MA) rats (Xu et al. 2010). It is worth noting that Dex can alleviate pancreatic injury and inflammatory response in mice with pancreatitis by inhibiting NLRP3 inflammasome (Li et al. 2018b). Yao et al. (2015) found that Dex can attenuate the acute kidney injury induced by autologous liver transplantation by inhibiting NF-κB pathway through α2A-AR subtype. Zhang et al. (2015) showed that Dex can down-regulate inflammatory factor levels in septic rats by inhibiting NF-κB pathway. Zhang et al. (2009) found in CFA-induced MA rats that intraperitoneal injection of Dex (20 μg kg^−1^) antagonized inflammatory pain in MA rats and suggested that Dex may be beneficial in the treatment of arthritic pain. However, the role and mechanism of Dex in the OA rat model is unclear. We induced a rat model of osteoarthritis by intra-articular injection of papain, and inter-catheter injection of Dex to intervene in the OA rat model to investigate the effect of Dex on pain and cartilage damage in OA rats.

## Materials and methods

### Animals and grouping

Specific-Pathogen-Free Wistar rats (Male, 280–320 g, 3-month-old) were purchased from Antai Kang Biotechnology (Beijing, China). Rats were kept under standard laboratory conditions (temperature 24 °C, 12 h light-dark cycle). They were fed standard diet and drank tap-water *ad libitum*. All experimental procedures were ethically approved by the Animal Use and Care Committee of the Lianyungang Oriental Hospital and were conducted in accordance with the National Institute for Health “Guide for the Care and Use of Laboratory Animals”.

### Model preparation and processing

The OA model was prepared according to the reference (Murat et al. 2007). A 4% papain solution (0.2 mL) and 0.1 mL of 0.03 mol L^−1^ l-cysteine were mixed and allowed to stand for 0.5 h. The mixture (20 μL) was injected into the right knee joint of the rat. Intra-articular injections were repeated on days 4 and 7. Rats in the control group (*n* = 14) were injected with the same amount of normal saline only in the right knee joint. The general health of rats was monitored as per the ethical guidelines of the research team’s institution. After the last injection of papain solution for 1 week and 2 weeks, four rats were randomly selected from OA and control group, and the paraffin sections of articular cartilage were obtained for Hematoxylin-eosin (HE) staining and Safranin O-Fast Green staining for model identification.

OA rats were randomly divided into four groups, 10 in each group: OA group, low dose Dex treatment group (L-DEX group), medium dose Dex treatment group (M-DEX group), high dose DEX treatment group (H-DEX group). The L-DEX, M-DEX and H-DEX groups were injected with 5, 10, or 20 μg kg^−1^ Dex into the right knee joint, respectively. Both the control group and the OA group were injected with the same volume of physiological saline, once a day, continuously for 4 weeks.

### Experimental design

Gait behavior tests were performed before modeling, and on days 0, 7, 14, 21, and 28 during Dex treatment, and the mechanical contraction threshold, heat-shrinking response latency, and the knee diameter were measured, simultaneously.

After the completion of DEX treatment, the rats were anesthetized by intraperitoneal injection of 1% sodium pentobarbital (35 mg kg^−1^), and sacrificed by cervical dislocation. Collect articular cartilage tissue, a portion was stored at −80 °C for enzyme-linked immunosorbent assay (ELISA) and western blot analysis, a portion was fixed in 4% paraformaldehyde overnight, and paraffin-embedded (5 μm) for histopathological analysis.

### Gait behavior test

The gait behavior of rats was semi-quantitatively analyzed between 0 and 4 points (Ma and Liang 2017). Rats walked on a 17 cm s^−1^ electric treadmill for 20 s. If the rat refuses to walk on the treadmill, it was recorded as 0; Rats walked 30% of the prescribed time, and the gait showed severe disharmony, which was recorded as 1 point; Rats walked 60% of the prescribed time, and the gait showed moderate disharmony, which was recorded as 2 points; Rats walked 90% of the prescribed time, and the gait showed a slight inconsistency, which was recorded as 3 points; When the rats have completed the prescribed time and the gait is normal, they are recorded as 4 points.

### Mechanical withdrawal response threshold detection

The rat paw withdrawal mechanical threshold (PWMT) was determined using a Ugo Basile thermal pain tester (Ugo Basile 37370, Comerio, Italy). All rats were placed in an acrylic cage with a mesh floor to accommodate the environment for 15 min. The probe of the tester was aimed at the right sole and stimulated for 3–6 s to observe whether the rat had a contraction reaction. A positive reaction occurs if the rat rapidly contracts and licks or shakes the foot during the stimulation time or when removing the filament. The minimum fiber stimulating force that produces a positive reaction is recorded as PWMT. Each rat was averaged three times, with an interval of 5 min.

### Heat shrinking foot reaction latency

The rat paw withdrawal thermal latency (PWTL) was determined using a Ugo Basile thermal pain tester. The rats were placed in a test chamber with a glass bottom and adapted to the environment for 15 min. Adjust the intensity of the infrared radiation probe of the tester, so that the ability to stimulate the rat was about 10 s, and the cutting time was set to 25 s. Rats with elevated or lame behavior were considered positive. The shortest time to produce a positive reaction is the PWTL. Each rat was averaged three times, with an interval of 5 min.

### Knee diameter evaluation

A precision tape measure was used to analyze knee diameter. The tape was positioned medio-laterally in the join interline region to quantify the joint diameter (mm) of the knee. Each rat was averaged three times.

### HE staining

The paraffin sections were dewaxed, graded alcohol hydrated, hematoxylin solution stained for 15 min, and eosin solution counterstained for 5 min. After dehydration, transparency and sealing of the gradient alcohol, the pathological condition of articular cartilage was observed by Image-Pro image analysis software.

### Safranin O-Fast green staining

After paraffin section was dewaxed and graded with alcohol, it was stained with 0.5% Fast Green for 20 min, stained with 0.5% Safranin O for 5 min, and then subjected to gradient alcohol dehydration, xylene transparent, neutral gum seal. The articular cartilage tissue was observed under a microscope.

### Mankin scores

After the Safranin O-Fast Green staining was completed, the degree of articular cartilage lesions was scored by two independent observers according to the modified Mankin scoring principle (Murat et al. 2007). The score range is 0–14, and the higher the score, the more severe the joint degeneration.

### Immunohistochemical analysis

Paraffin sections (5 μm) were dehydrated, hydrated with gradient alcohol, incubated in citrate buffer for 15 min at 95 °C, and incubated with peroxidase blocker for 30 min. The primary antibodies of MMP-13 and Collagen II were incubated overnight at 4 °C. Sections incubated with PBS served as negative controls. The FITC-labeled secondary antibody was incubated for 1 h, and after washing with PBS, the DAB solution was developed. After washing with tap water, it is counterstained with hematoxylin, dehydrated with gradient alcohol, and transparent with xylene, and then sealed with a neutral gum. Five sections of each slice were taken and photographed. The mean optical density (MOD) of immunohistochemistry was quantified by two individuals following a double-blind principle.

### Enzyme-linked immunosorbent assay（ELISA）

The levels of IL-1β, IL-18, TNF-α and IL-6 in cartilage were detected by enzyme-linked immunosorbent assay (ELISA). The ELISA kit was purchased from Anorui Kang (Tianjin, China). All operations are carried out in strict accordance with the manufacturer’s instructions.

### Western blot analysis

Knee articular cartilage tissue was homogenized in a lysate containing protease inhibitors and nuclear proteins and cytosolic proteins were extracted. The protein concentration of each sample was determined using a BCA Protein Assay Kit (Pierce, USA). Total protein (30 μg) was separated by sodium dodecyl sulfate-polyacrylamide gel electrophoresis and transferred to a polyvinylidene fluoride membrane (Millipore, USA). After blocking the 5% delipidated protein for 1 h, the corresponding primary antibody of MMP-13, Collagen II, NLRP3, ASC, caspase-1 p10, NF-κB p65, IκB was added and incubated overnight at 4 °C. After washing, horseradish peroxidase-labeled secondary antibody (1:2000, Cell Signaling, USA) was added and incubated for 1.5 h at room temperature. After adding the luminescent liquid, a photo was taken with a gel imager. Using β-actin and histone as internal parameters, the relative expression levels of the proteins were calculated by statistical gray value.

### Statistical analyses

SPSS software (ver. 07 for Windows; SPSS Inc., Chicago, IL). The results were represented as mean ± SD. Comparative studies were analyzed by Student’s *t*-test or one- or two-way ANOVA test followed by *post hoc* test with *p* values less than 0.05 considered as statistically significant.

## Results

### Identification of OA rat model

After injection of 20 μL of 4% papain solution into the right knee of the rats, about 80% of the rats were observed to have different degrees of joint swelling and activity limitation on the 5th day after papain injection. On day 7, all abnormalities were observed in all model rats. The model was identified by HE staining and Safranin O-Fast Green staining at 1 week and 2 weeks after the last papain injection.

**Figure 1.**
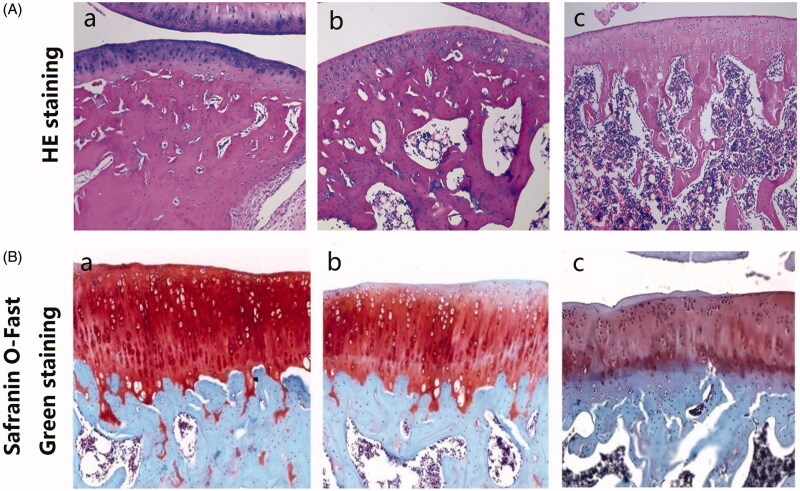
Identification of a rat model of OA induced by intra-articular injection of papain. (A) The HE staining of articular cartilage tissue of the rats in the control group (a), and the 1 week (b) or 2 weeks (c) after the last injection of papain (×100). (B) The safranin O-Fast Green staining of articular cartilage tissue of the rats in the control group (a), and the 1 week (b) or 2 weeks (c) after the last injection of papain (×200). Rats in the 1 week (b) or 2 weeks (b) after the last papain injection groups were injected with 4% papain solution (20 μL) in the right knee joint. Rats in the control group were injected with the same amount of normal saline only.

HE staining revealed (Figure 1(A)) that the control cartilage had a clear structure, no defect area, and the chondrocytes were arranged neatly (Figure 1(Aa)). Fractures and small ulcers were observed on the cartilage surface of OA rats 1 week after papain injection, and the four-layer structure was blurred and the number of chondrocytes was significantly reduced (Figure 1(Ab)). After 2 weeks of papain injection, cartilage damage was increased in OA rats (Figure 1(Ac)).

Safranin O-Fast Green staining also showed moderate saffron staining in OA rats 1 week after papain injection (Figure 1(Bb)), and severe cartilage destruction occurred 2 weeks after injection (Figure 1(Bc)). With the prolongation of postoperative time, cartilage destruction showed a significant tendency to aggravate. These results confirmed the successful preparation of the OA rat model.

### The effect of Dex on pain behavior in OA rats

**Figure 2.**
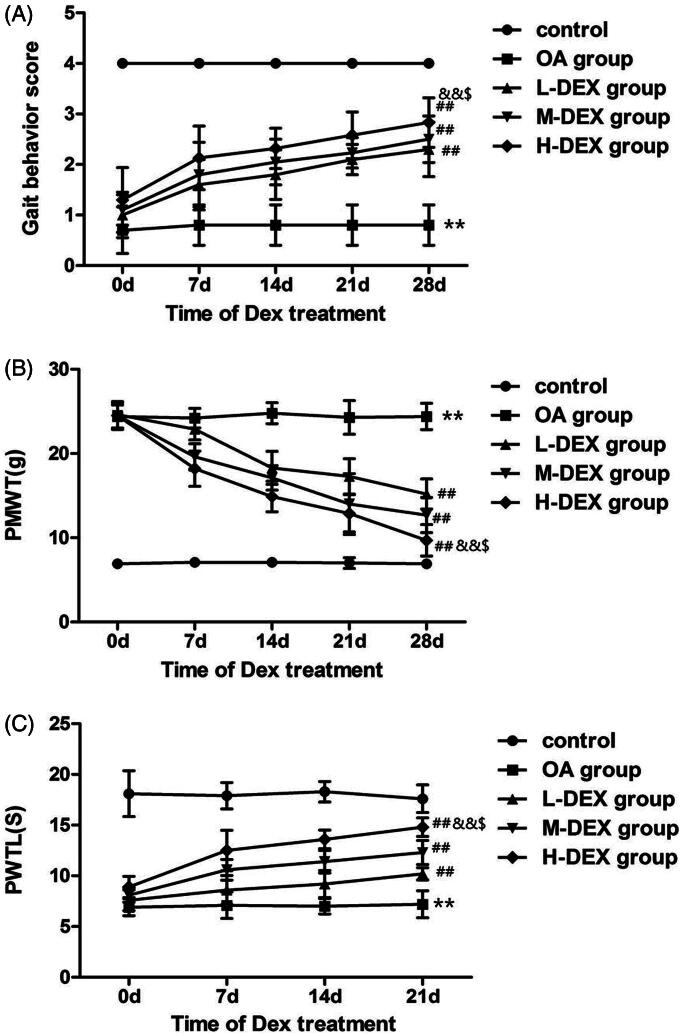
Effect of Dex on pain behavior in OA rats. Gait behavior test (A), mechanical withdrawal response threshold test (B) and heat-shrinking foot reaction latency test(C) on day 0, 7, 14, 21 and 28 during intra-articular injection of Dex to analyze the improvement of pain behavior in OA rats by Dex. Values are means ± SD. *N* = 10. **p < 0.01 vs. control group; ^##^*p < *0.01 vs. OA group; ^&&^*p < *0.01 vs. L-DEX group; ^$^*p < *0.05 vs. M-DEX group.

To analyze the therapeutic effect of Dex on OA rats, gait behavioral tests were performed on days 0, 7, 14, 21, and 28 during Dex treatment to analyze spontaneous pain behavior in OA rats (Figure 2(A)). We found that the gait behavior score of OA rats was significantly higher than that of the control group (*p* < 0.05), suggesting that intra-articular injection of papain can cause pain behavior in OA rats. On the 28th day of Dex treatment, the gait behavior scores of OA rats in L-DEX group, M-DEX group and H-DEX group were significantly higher than those of OA group (*p* < 0.05), suggesting that Dex treatment can improve joint pain in OA rats in a dose-dependent manner.

PWMT and PWTL are commonly used indicators of reactive neuropathic pain and are inversely related to the degree of neuropathic pain (Ma and Liang 2017). We evaluated the effects of Dex on neuropathic pain in OA rats by analyzing PWMT and PWTL (Figure 2(B,C)). The results showed that the PWMT and PWTL values of OA rats showed an increasing trend during Dex treatment. With the prolongation of time and the increase of dose, the effect of Dex on relieving pain in OA rats gradually became obvious. On the 28th day of Dex treatment, the PWMT and PWTL of L-DEX group, M-DEX group and H-DEX group were significantly lower than those of OA group (*p* < 0.05), indicating that DEX has an analgesic effect on arthritis.

### Effect of Dex on the morphology and structure of articular cartilage in OA rats

**Figure 3.**
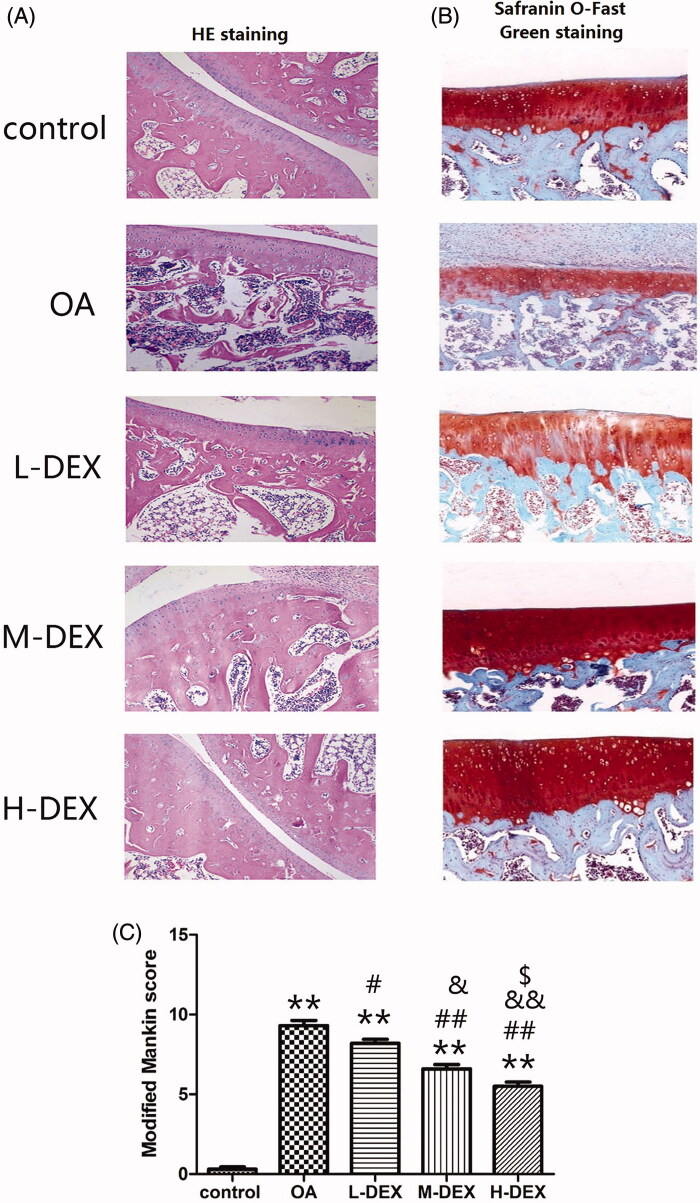
Effect of Dex on the morphological structure of articular cartilage in OA rats. After Dex treatment was completed, HE staining (A, ×100) and Safranin O staining (B, ×200) were used to analyze the morphological structure of cartilage tissue of OA rats. (C) Quantitative analysis of cartilage lesions in OA rats using a modified Mankin score. Values are means ± SD. *N* = 10. **p < 0.01 vs. control group; ^#^*p < *0.05, ^##^*p < *0.01 vs. OA group; ^&^*p < *0.05, ^&&^*p < *0.01 vs. L-DEX group; ^$^*p < *0.05 vs. M-DEX group.

After Dex treatment was completed, we analyzed the morphological structure of cartilage tissue of OA rats by HE staining (Figure 3(A)) and Safranin O-Fast Green staining (Figure 3(B)). The normal cartilage in the control group was stratified, and the chondrocytes in each layer were arranged neatly and the matrix was evenly distributed. In the OA group, the cartilage surface was damaged, the cartilage tissue was largely detached and the structure was blurred, and the chondrocytes were disorderly arranged, accompanied by lymphocyte infiltration. Safranin O-Fast Green staining analysis also found that the cartilage matrix of the control group was uniformly stained (red), the bone tissue was green and the layers were distinct; while the cartilage matrix of the OA group became lighter, and the boundary between the cartilage matrix and the bone tissue was blurred. Pathological injury of cartilage tissue of OA rats can be improved by 4 weeks treatment of joint cavity injection of Dex.

The Mankin score can quantify the degree of cartilage damage by articular cartilage structure, chondrocyte number distribution, cartilage matrix coloration, and tidal line (Murat et al. 2007). We used a modified Mankin score to analyze (Figure 3(C)) and found that, compared with the control group, the cartilage tissue score of the OA group was significantly higher (*p* < 0.05). The Mankin scores of the M-DEX group and the H-DEX group were significantly lower than those of the OA group (*p* < 0.05). These results suggested that Dex treatment can improve the pathological damage of articular cartilage in OA rats and reduce the degree of articular cartilage degeneration in OA rats.

### Effect of Dex on the metabolism of cartilage matrix in OA rats

**Figure 4.**
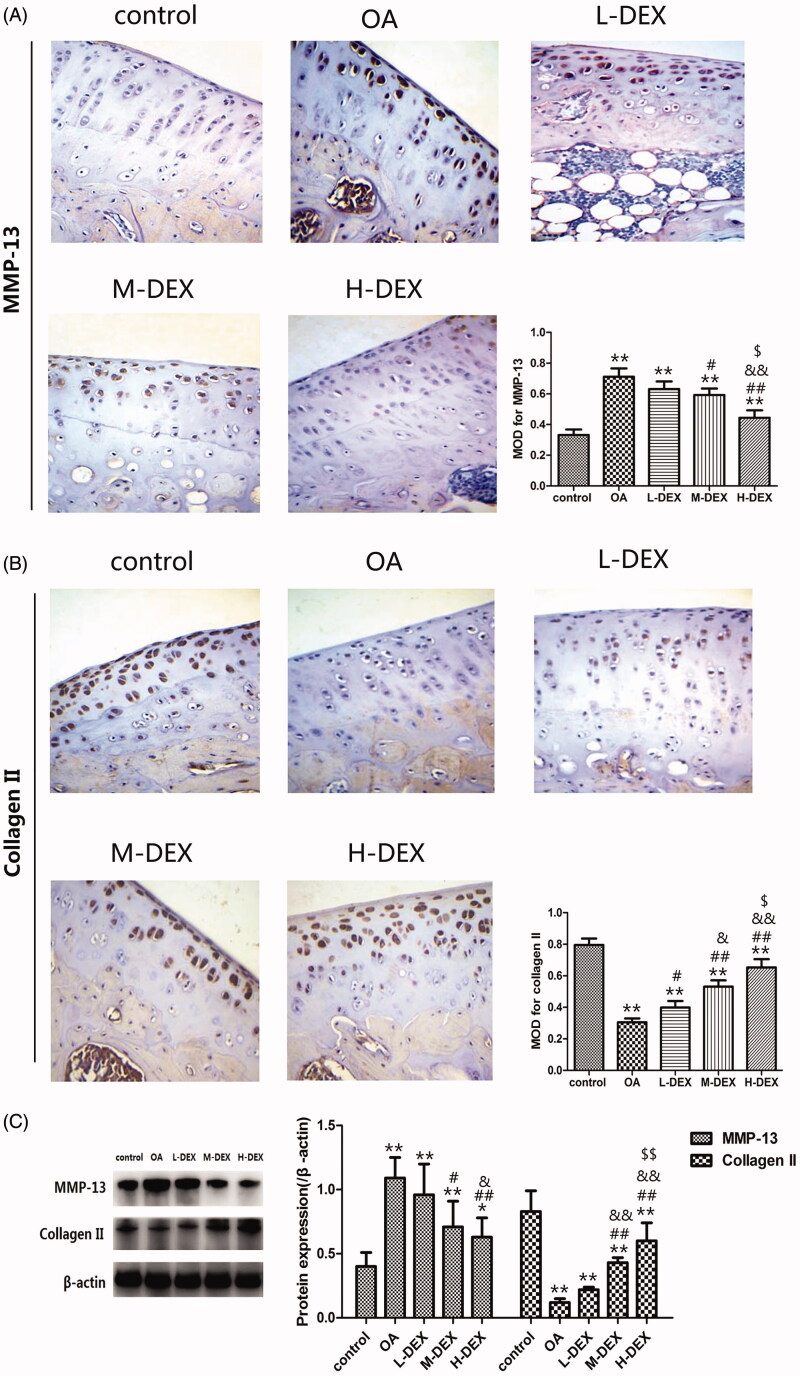
Effect of Dex on cartilage matrix metabolism in OA rats. (A) The expression of MMP-13 and collagen II expression in cartilage tissue was analyzed by immunohistochemistry after Dex treatment (×400). (B) Western blot analysis of expression of MMP-13 and collagen II in cartilage tissue. Values are means ± SD. *N* = 10. **p < 0.01 vs. control group; ^#^*p < *0.05, ^##^*p < *0.01 vs. OA group; ^&^*p < *0.05, ^&&^*p < *0.01 vs. L-DEX group; ^$^*p < *0.05, ^$$^*p < *0.01 vs. M-DEX group.

The imbalance of synthesis and degradation of cartilage matrix is an important cause of OA cartilage degradation, in which MMPs play a decisive role (Lin et al. 2018). To analyze the effect of Dex on cartilage matrix metabolism, we used immunohistochemistry to analyze the expression of MMP-13 and collagen II in cartilage tissue. Immunohistochemical analysis revealed that (Figure 4(A)), compared with the control group, the positive expression of MMP-13 protein in the articular cartilage tissue of the OA group was enhanced, while the positive expression of collagen II was weakened (*p* < 0.05). Compared with the OA group, the positive expression of MMP-13 protein in the cartilage tissue of the Dex group was weakened, and the positive expression of collagen II was enhanced (*p* < 0.05).

Western blot analysis also showed that compared with the OA group, the protein expression of MMP-13 in the cartilage tissues of the M-DEX group and the H-DEX group was significantly decreased, while the expression level of collagen II was significantly increased (*p* < 0.05, Figure 4(B)). These results revealed that the improvement of Dex on articular cartilage degradation in OA rats may be related to the regulation of cartilage matrix metabolism.

### Effect of Dex on inflammatory response of cartilage tissue in OA rats

**Figure 5.**
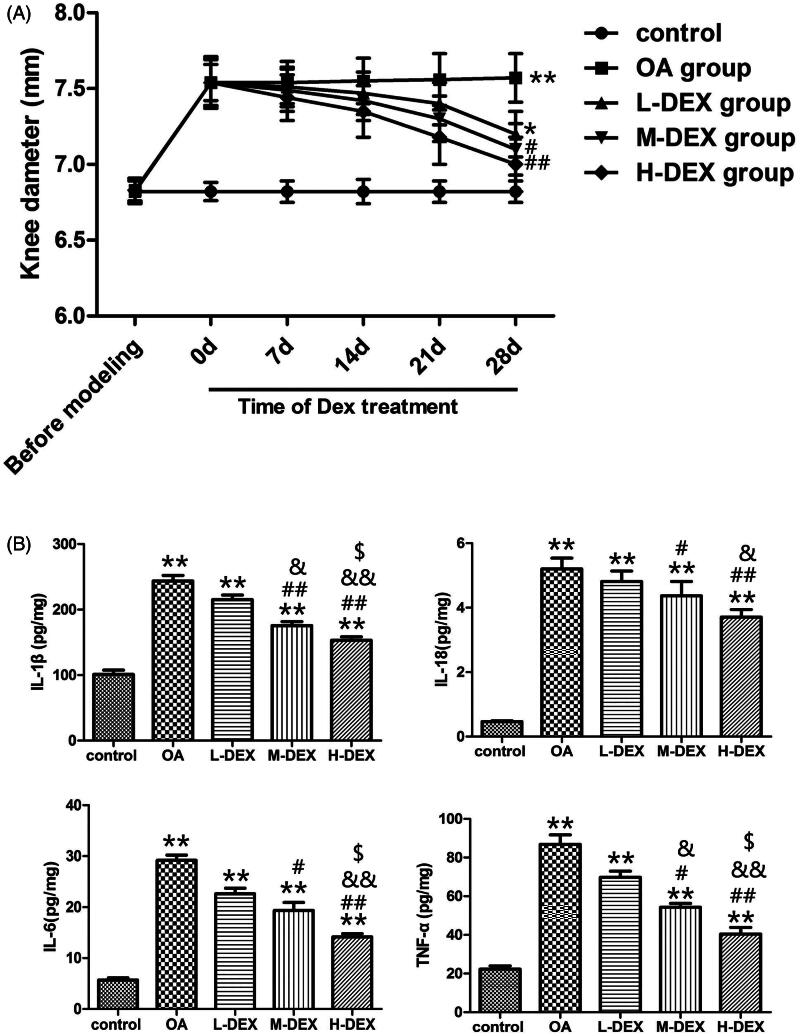
Effect of Dex on inflammatory response in OA rats. (A) Joint diameters of OA rats were measured on days 0, 7, 14, 21, and 28 during intra-articular injection of Dex. (B) The content of IL-1β, IL-18, IL-6 and TNF-α in cartilage tissues was detected by ELISA after Dex treatment. Values are means ± SD. *N* = 10. **p < 0.01 vs. control group; ^#^*p < *0.05, ^##^*p < *0.01 vs. OA group; ^&^*p < *0.05, ^&&^*p < *0.01 vs. L-DEX group; ^$^*p < *0.05 vs. M-DEX group.

To indirectly evaluate the joint inflammation (edema and cellular infiltrate), we measured the knee diameter (Figure 5(A)). There was no significant difference in joint diameter between the groups before the model establishment. After model establishment (0 d of treatment with Dex), the joint diameter of OA group, L-DEX group, M-DEX and H-DEX group was significantly higher than that of the control group (*p* < 0.05), suggesting that papain injection can cause joint swelling in rats, and the inflammatory reaction is involved in the development of OA. On the 28th day of Dex treatment, the joint diameters of the M-DEX group and the H-DEX group were significantly lower than those of the OA group (*p* < 0.05). It was suggested that the therapeutic effect of Dex on OA rats may be related to its anti-inflammatory effect.

Proinflammatory factors are important mediators of the inflammatory response. Abnormal secretion of inflammatory factors can cause abnormal metabolism of cartilage matrix and increase of apoptotic cells, which eventually leads to degeneration of cartilage (Lisignoli et al. 2004). To analyze the anti-inflammatory effects of Dex on OA rats, we used ELISA to detect the levels of inflammatory mediators such as IL-1β, IL-18, IL-6 and TNF-α (Figure 5(B)). We found that the levels of IL-1β, IL-18, IL-6 and TNF-α in the OA group were significantly higher than those in the control group (*p* < 0.05). After the treatment of Dex, the levels of IL-1β, IL-18, IL-6 and TNF-α in M-DEX group and H-DEX group were significantly lower than those in OA group (*p* < 0.05), suggesting that Dex can reduce the secretion of inflammatory factors in cartilage tissue of OA rats.

### Effect of Dex on NLRP3 inflammasome in OA rats

**Figure 6.**
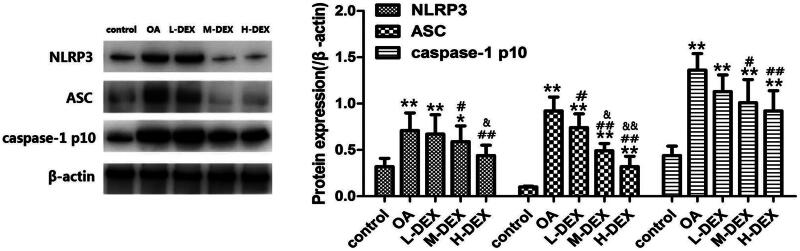
Effect of Dex on NLRP3 inflammatory bodies in OA rats. The expression levels of NLRP3, ASC and caspase-1 in cartilage tissues were detected by Western blotting. Values are means ± SD. *N* = 10. **p < 0.01 vs. control group; ^#^*p < *0.05, ^##^*p < *0.01 vs. OA group; ^&^*p < *0.05, ^&&^*p < *0.01 vs. L-DEX group.

The secretion of IL-1β and IL-18 is mainly regulated by activated caspase-1, and NLRP3 inflammasome activation is a key regulator of caspase-1 activation (Allen et al. 2009). To investigate the anti-inflammatory mechanism of DEX in improving cartilage damage in OA rats, we further analyzed the activity of NLRP3 inflammasome in articular cartilage (Figure 6). We found that the expression of NLRP3, ASC and caspase-1 p10 in articular cartilage tissue of OA group was significantly increased compared with the control group (*p* < 0.05). The expression of NLRP3, ASC and caspase-1 p10 in M-DEX group and L-DEX group was significantly lower than that of OA group (*p* < 0.05).

### Effect of Dex on NF-κB pathway

**Figure 7.**
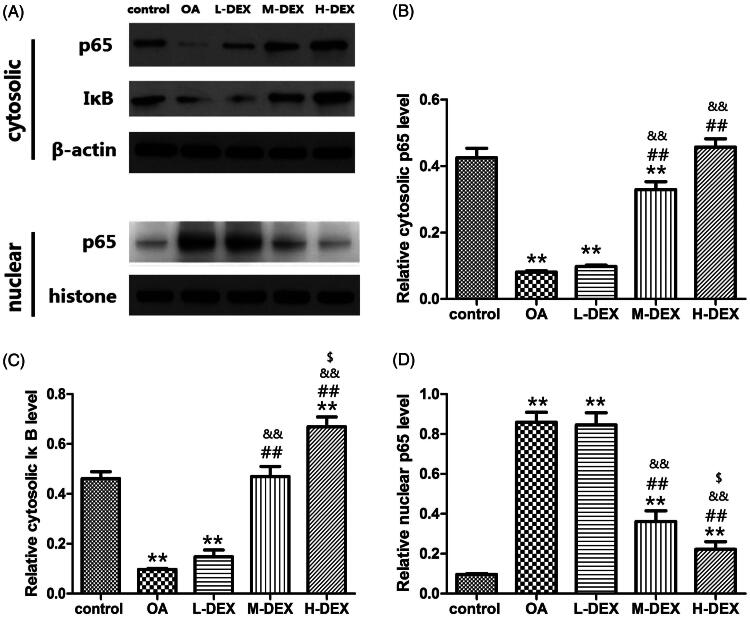
Dex inhibits nuclear translocation of NF-κB in articular cartilage tissue of OA rats. (A) NF-κB p65 in cytoplasm and nucleus, and IκB levels in cytoplasm were analyzed by western blot. (B) The expression of NF-κB p65 in cytoplasm. (C) The expression of IκB in cytoplasm. (D) The expression of NF-κB p65 in nucleus. Dex treatment significantly inhibited the degradation of IκB and the nuclear translocation of p65 in cartilage tissues of OA rats. Values are means ± SD. *N* = 10. **p < 0.01 vs. control group; ^##^*p < *0.01 vs. OA group; ^&&^*p < *0.01 vs. L-DEX group; ^$^*p < *0.05 vs. M-DEX group.

As the most important transcription factor, NF-κB is closely related to inflammatory reaction, cartilage degradation and matrix metabolism during the progression of OA (Roman-Blas and Jimenez 2006). p65 is an important functional subunit of NF-κB, and activated NF-κB plays a regulatory role mainly by translocation of p65 into the nucleus. We further analyzed the effect of Dex on the NF-κB pathway in cartilage tissue. As shown in Figure 7, the p65 protein level in the nuclei of the control rats was low. OA can induce the translocation of p65 from the cytoplasm to the nucleus to increase the content of p65 protein in the nucleus, which may be related to the decrease of IκB level in the cytoplasm. While Dex treatment can significantly inhibit the degradation of IκB and nuclear translocation of p65, suggesting that it may reduce the secretion of inflammatory factors by inhibiting the activation of NF-κB.

## Discussion

Injection of drugs into the knee joint cavity is known as the classical method for establishing animal models of OA (Kalbhen 1987). Injection of papain is one of the most common drugs, which has the advantages of high model success rate, short modeling time and good repeatability, and has similar characteristics to human OA (Murat et al. 2007). We constructed a rat OA model by injecting papain into the knee joint cavity and observed the pain behavior of the model rats, the cartilage tissue damage of the knee joint, and the severe inflammatory reaction, confirming the successful modeling. As a selective α2AR agonist, Dex has been widely used in adult intensive care units and clinical anesthesia (Bhana et al. 2000). Dex has been shown to have significant analgesic effects in neuropathic pain and chronic inflammatory pain models (Xu et al. 2010), including osteoarthritic rats (Li et al. 2016). We treated OA rats by intra-articular injection of Dex as an intervention, which not only increased the local drug concentration, but also reduced systemic adverse reactions (Cheng et al. 2012). We found that continuous treatment of Dex for 4 weeks can effectively increase the pain threshold of OA rat model, reduce the joint diameter and Makin score of OA rats, suggesting that Dex has a therapeutic effect on OA and can improve joint inflammation and tissue damage.

Although the pathogenesis of OA has not been fully elucidated, a large number of studies have confirmed that the inflammatory response is closely related to the progression of OA (Haywood et al. 2003). IL-1β and TNF-α are recognized as pathogenic factors in synovial inflammation and cartilage injury (Lisignoli et al. 2004). TNF-α not only synergizes with IL-1β, but also induces IL-6 production, which plays a decisive role in the occurrence and development of OA (Guler-Yuksel et al. 2010). IL-1β is the most important cytokine in the pathological process of OA that can further induce the expression of other pro-inflammatory proteins and expand the inflammatory response, which ultimately aggravates the destruction of articular cartilage (Margerie et al. 1997; Ji et al. 2016; Wang et al. 2016). Scholars have suggested that the main mechanism of action of IL-1β on the degradation of articular cartilage is to promote the degradation of articular cartilage matrix (Ji et al. 2016). Up-regulation of IL-1β-induced expression of matrix metalloproteinases (MMPs), especially up-regulation of MMP-13, is an important factor in the irreversible destruction of cartilage matrix (Vincenti and Brinckerhoff 2001). Moreover, IL-1β binds to its receptor can activate the NF-κB pathway (Zhong et al. 2013). Inhibition of the release of inflammatory factors is one of the important means to improve osteoarthritis pain and treat osteoarthritis (Xia 2010; Lin et al. 2016; Nixon et al. 2018).

In addition to analgesic and sedative effects, Dex has been widely confirmed for its anti-inflammatory effects. Taniguchi et al. (2008) believe that a possible mechanism by which Dex inhibits inflammatory response is that Dex regulates cytokine production, as their study shows that Dex can significantly inhibit the production of pro-inflammatory factors such as IL-6 and TNF-α in endotoxemia mice. Other studies have also shown similar results (Memiş et al. 2007; Qiao et al. 2009). Memiş et al. (2007) also found in critically ill patients that Dex can regulate the production of TNF-α, IL-6, IL-1β to exhibit anti-inflammatory effects. We found in papain-induced OA rats that different doses of Dex could improve joint swelling in OA rats and reduce the content of inflammatory factors in cartilage tissue. Simultaneously, Dex can decrease the expression of MMP-13 in cartilage tissue of OA rats and increase the content of type II collagen. We hypothesize that Dex may inhibit cartilage matrix degradation by inhibiting the release of inflammatory cytokines in cartilage tissue to improve cartilage damage.

Inflammasome is a protein complex found in the cytoplasm that relies on the molecular platform of caspase-1 to promote the maturation and release of IL-1β and IL-18 to cause an inflammatory response (Allen et al. 2009). NLRP3 inflammasome is the most studied NLR family member that composed of NLRP3, apopto-associated speck-like protein containing acupaspase recruitment domain (ASC) and pro-caspase-1 (Zhou et al. 2011). NLRP3 can be activated by a variety of pathogen-associated molecular patterns (PAMPs) or danger-associated molecular patterns (DAMPs). These stimulation signals also up-regulate the expression of NLRP3 by activating the NF-κB pathway (Bauernfeind et al. 2009). Caspase-1 is an effector protein of NLRP3 inflammasome. Once caspase-1 is activated, inactive pro-IL-1β and IL-18 can be shear processed into mature IL-1β and IL-18. Recent studies have confirmed that inflammatory pathways mediated by NLRP3 inflammasome are closely related to the occurrence of OA (McAllister et al. 2018). We also observed activation of NLRP3 inflammasome in articular cartilage tissue of OA rats. Li H et al. (2018a) revealed that Dex inhibits inflammation in microglia cells under stimulation of LPS and ATP via NLRP3/caspase-1 cascades, which adds new understanding of the anti-inflammatory mechanism of DEX. Li Y et al. (2018b) also proposed Dex attenuates pancreatic injury and inflammatory response in mice by reducing NLRP3 activation. Additionally, the anti-inflammatory effects of Dex are also associated with inhibition of NLRP3 inflammasome activation in animal models of various inflammatory diseases such as brain injury (Yin et al. 2018) and lung injury (Zhang et al. 2016). We treated OA rats by intra-articular injection of Dex and found that Dex can significantly inhibit the activation of NLRP3 inflammasome in cartilage tissue and its downstream effects.

IL-1β is not only a downstream molecule of the NLRP3 inflammasome, but also an upstream molecule of the NF-κB pathway in complex inflammatory molecular networks (Zhu et al. 2014). Moreover, NF-κB plays an important role in the transcription and expression of inflammatory molecules (IL-1β, IL-6, TNF-α, PGE2) genes (Chen et al. 2013). Qiao et al. (2012) found that NLRP3 expression can be induced in an NF-κB-dependent manner. Zhong et al. (2013) demonstrated that IL-1β promotes the NF-κB p65 translocation from the cytoplasm to the nucleus by increasing the degradation of IκBα, then binds to the target gene promoter region of MMPs and iNOS. Activation of the NF-κB pathway in chondrocytes induces synovitis and joint destruction by causing the production of pro-inflammatory mediators and metabolic mediators, particularly IL-6, IL-8, and MMPs (Chen et al. 2013). Besides, NF-κB can also function with other transcription factors such as activator protein-1 (AP-1). In synovial cells stimulated by IL-1β or TNF-α, activated AP-1 and NF-κB synergistically stimulate the production of MMPs to cause destruction of bone and articular cartilage (Grall et al. 2003). Toegel et al. (2012) concluded that abolishing NF-κB (p65/p50)-driven MMP-13 promoter activation inhibits IL-1β-mediated upregulation of MMP-13 mRNA and protein levels. Tomita et al. (1999) used antisense RNA technology to block the transfection activity of NF-κB to reduce the inflammation of arthritis. However, Lv et al. (2018) treated human liver cells with different concentrations of Dex *in vitro*, and found that promoted effect on liver regeneration and liver function recovery through inhibiting NLRP3 inflammasome activation not NF-κB pathway. We found in OA rats that Dex can not only reduce the inflammatory response by regulating NLRP3-ASC-caspase-1-IL-1β/IL-18 axis to improve cartilage matrix degradation, but also inhibit the nuclear translocation of NF-κB in cartilage tissue, which may be related to the different ways in which OA is modeled. We hypothesized that Dex can ultimately reduce cartilage damage in OA rats by inhibiting NF-κB and NLRP3 inflammasome-mediated inflammation. However, the interaction between NF-κB and NLRP3 inflammasome needs further investigation.

## Conclusions

Dex can improve pain symptoms and cartilage tissue damage in OA rats. The protective effect of Dex on the OA rat model may be related to the inhibition of the activation of NF-κB and NLRP3 inflammasome to reduce the maturation and release of inflammatory factors in cartilage tissue. However, we only initially explored the effect of Dex on the inflammatory response in OA rats. The pathophysiological process of OA is complex, and the protective effect and related mechanism of Dex on OA remains to be further explored.

## Data Availability

All data generated or analyzed during this study are included in this published article.

## References

[CIT0001] Allen IC, Scull MA, Moore CB, Holl EK, McElvania-TeKippe E, Taxman DJ, Guthrie EH, Pickles RJ, Ting JP. 2009. The NLRP3 inflammasome mediates *in vivo* innate immunity to influenza A virus through recognition of viral RNA. Immunity. 30(4):556–565. 19362020 10.1016/j.immuni.2009.02.005 PMC2803103

[CIT0002] Bauernfeind FG, Horvath G, Stutz A, Alnemri ES, MacDonald K, Speert D, Fernandes-Alnemri T, Wu J, Monks BG, Fitzgerald KA. 2009. Cutting edge: NF-kappaB activating pattern recognition and cytokine receptors license NLRP3 inflammasome activation by regulating NLRP3 expression. J Immunol. 183(2):787–791. 19570822 10.4049/jimmunol.0901363 PMC2824855

[CIT0003] Benito MJ, Veale DJ, FitzGerald O, van den Berg WB, Bresnihan B. 2005. Synovial tissue inflammation in early and late osteoarthritis. Ann Rheum Dis. 64(9):1263–1267. 15731292 10.1136/ard.2004.025270 PMC1755629

[CIT0004] Bhana N, Goa KL, McClellan KJ. 2000. Dexmedetomidine. Drugs. 59(2):263–268. discussion 269–70. 10730549 10.2165/00003495-200059020-00012

[CIT0005] Bougault C, Gosset M, Houard X, Salvat C, Godmann L, Pap T, Jacques C, Berenbaum F. 2012. Stress-induced cartilage degradation does not depend on the NLRP3 inflammasome in human osteoarthritis and mouse models. Arthritis Rheum. 64(12):3972–3981. 22933232 10.1002/art.34678

[CIT0006] Buckwalter JA, Mankin HJ. 1998. Articular cartilage: degeneration and osteoarthritis, repair, regeneration, and transplantation. Instr Course Lect. 47:487–504. 9571450

[CIT0007] Chen J, Crawford R, Xiao Y. 2013. Vertical inhibition of the PI3K/Akt/mTOR pathway for the treatment of osteoarthritis. J Cell Biochem. 114(2):245–249. 22930581 10.1002/jcb.24362

[CIT0008] Cheng OT, Souzdalnitski D, Vrooman B, Cheng J. 2012. Evidence-based knee injections for the management of arthritis. Pain Med. 13(6):740–753. 22621287 10.1111/j.1526-4637.2012.01394.x PMC3376243

[CIT0009] Grall F, Gu X, Tan L, Cho JY, Inan MS, Pettit AR, Thamrongsak U, Choy BK, Manning C, Akbarali Y, et al. 2003. Responses to the proinflammatory cytokines interleukin-1 and tumor necrosis factor alpha in cells derived from rheumatoid synovium and other joint tissues involve nuclear factor kappaB-mediated induction of the Ets transcription factor ESE-1. Arthritis Rheum. 48(5):1249–1260. 12746898 10.1002/art.10942

[CIT0010] Guler-Yuksel M, Allaart CF, Watt I, Goekoop-Ruiterman YP, de Vries-Bouwstra JK, van Schaardenburg D, van Krugten MV, Dijkmans BA, Huizinga TW, Lems WF, et al. 2010. Treatment with TNF-α inhibitor infliximab might reduce hand osteoarthritis in patients with rheumatoid arthritis. Osteoarthr Cartil. 18(10):1256–1262. 10.1016/j.joca.2010.07.011 20691795

[CIT0011] Haywood L, McWilliams DF, Pearson CI, Gill SE, Ganesan A, Wilson D, Walsh DA. 2003. Inflammation and angiogenesis in osteoarthritis. Arthritis Rheum. 48(8):2173–2177. 12905470 10.1002/art.11094

[CIT0012] Ji Q, Xu X, Zhang Q, Kang L, Xu Y, Zhang K, Li L, Liang Y, Hong T, Ye Q, et al. 2016. The IL-1β/AP-1/miR-30a/ADAMTS-5 axis regulates cartilage matrix degradation in human osteoarthritis. J Mol Med. 94(7):771–785. 27067395 10.1007/s00109-016-1418-z

[CIT0013] Kalbhen DA. 1987. Chemical model of osteoarthritis – a pharmacological evaluation. J Rheumatol. 14:130–131. 3625668

[CIT0014] Lawrence RC, Felson DT, Helmick CG, Arnold LM, Choi H, Deyo RA, Gabriel S, Hirsch R, Hochberg MC, Hunder GG, et al. 2008. Estimates of the prevalence of arthritis and other rheumatic conditions in the United States. Part II. Arthritis Rheum. 58(1):26–35. 18163497 10.1002/art.23176 PMC3266664

[CIT0015] Li H, Ji F, Zhou Y, Zhou S, Ji D, Xu H. 2016. IFN-γ is involved in the analgesic effect of dexmedetomidine in a rat model of monoarthritis. Prog Modern Biomed. 16:5847–5850.

[CIT0016] Li H, Zhang X, Chen M, Chen J, Gao T, Yao S. 2018a. Dexmedetomidine inhibits inflammation in microglia cells under stimulation of LPS and ATP by C-FOS/NLRP3/CASPASE-1 cascades. Excli J. 17:302–311. 29743866 10.17179/excli2017-1018 PMC5938529

[CIT0017] Li Y, Pan Y, Gao L, Lu G, Zhang J, Xie X, Tong Z, Li B, Li G, Li W. 2018b. Dexmedetomidine attenuates pancreatic injury and inflammatory response in mice with pancreatitis by possible reduction of NLRP3 activation and up-regulation of NET expression. Biochem Biophys Res Commun. 495(4):2439–2447. 29269298 10.1016/j.bbrc.2017.12.090

[CIT0018] Lin H, Hay E, Latourte A, Funck-Brentano T, Bouaziz W, Ea HK, Khatib AM, Richette P, Cohen-Solal M. 2018. Proprotein convertase furin inhibits matrix metalloproteinase 13 in a TGFβ-dependent manner and limits osteoarthritis in mice. Sci Rep. 8(1):10488. 29992982 10.1038/s41598-018-28713-2 PMC6041273

[CIT0019] Lin P, Ma Y, Cheng H, Shao X, Zheng W, Zheng X, Li X, Ye H. 2016. Mechanism of Duhuo Jisheng decoction regulating inflammation and inhibiting cartilage degeneration of osteoarthritis. ArthritisRheum. 5:51–54.

[CIT0020] Lisignoli G, Cristino S, Toneguzzi S, Grassi F, Piacentini A, Cavallo C, Facchini A, Mariani E. 2004. IL1beta and TNFalpha differently modulate CXCL13 chemokine in stromal cells and osteoblasts isolated from osteoarthritis patients: evidence of changes associated to cell maturation. Exp Gerontol. 39(4):659–665. 15050303 10.1016/j.exger.2003.09.030

[CIT0021] Lv M, Zeng H, He Y, Zhang J, Tan G. 2018. Dexmedetomidine promotes liver regeneration in mice after 70% partial hepatectomy by suppressing NLRP3 inflammasome not TLR4/NFkappaB. Int Immunopharmacol. 54:46–51. 29100037 10.1016/j.intimp.2017.10.030

[CIT0022] Ma W, Liang Y. 2017. An experimental research on the analgesis effect and underlying mechanism of BTX-A for rat knee osteoarthritis. Chin J Rehabil Med. 32:649–653.

[CIT0023] Margerie D, Flechtenmacher J, Büttner F, Karbowski A, Puhl W, Schleyerbach R, Bartnik E. 1997. Complexity of IL-1β induced gene expression pattern in human articular chondrocytes. Osteoarthritis Cartilage. 5(2):129–138. 9135824 10.1016/s1063-4584(97)80006-4

[CIT0024] McAllister MJ, Chemaly M, Eakin A, Gibson D, McGilligan V. 2018. NLRP3 as a potentially novel biomarker for the management of osteoarthritis. Osteoarthr Cartil. 26(5):612–619. 10.1016/j.joca.2018.02.901 29499288

[CIT0025] McCarthy GM, Cheung HS. 2009. Point: hydroxyapatite crystal deposition is intimately involved in the pathogenesis and progression of human osteoarthritis. Curr Rheumatol Rep. 11(2):141–147. 19296887 10.1007/s11926-009-0020-6

[CIT0026] Memiş D, Hekimoğlu S, Vatan I, Yandim T, Yüksel M, Süt N. 2007. Effects of midazolam and dexmedetomidine on inflammatory responses and gastric intramucosal pH to sepsis, in critically ill patients. Br J Anaesth. 98(4):550–552. 17363413 10.1093/bja/aem017

[CIT0027] Mridha AR, Wree A, Robertson AAB, Yeh MM, Johnson CD, Van Rooyen DM, Haczeyni F, Teoh NC-H, Savard C, Ioannou GN, et al. 2017. NLRP3 inflammasome blockade reduces liver inflammation and fibrosis in experimental NASH in mice. J Hepatol. 66(5):1037–1046. 28167322 10.1016/j.jhep.2017.01.022 PMC6536116

[CIT0028] Murat N, Karadam B, Ozkal S, Karatosun V, Gidener S. 2007. Quantification of papain-induced rat osteoarthritis in relation to time with the Mankin score. Acta Orthop Traumatol Turc. 41(3):233–237. 17876125

[CIT0029] Nixon AJ, Grol MW, Lang HM, Ruan MZC, Stone A, Begum L, Chen Y, Dawson B, Gannon F, Plutizki S, et al. 2018. Disease‐modifying osteoarthritis treatment with interleukin‐1 receptor antagonist gene therapy in small and large animal models. Arthritis Rheumatol. 70(11):1757–1768. 30044894 10.1002/art.40668

[CIT0030] Qiao H, Sanders RD, Ma D, Wu X, Maze M. 2009. Sedation improves early outcome in severely septic Sprague Dawley rats. Crit Care. 13(4):R136. 19691839 10.1186/cc8012 PMC2750194

[CIT0031] Qiao Y, Wang P, Qi J, Zhang L, Gao C. 2012. TLR-induced NF-κB activation regulates NLRP3 expression in murine macrophages. FEBS Lett. 586(7):1022–1026. 22569257 10.1016/j.febslet.2012.02.045

[CIT0032] Ramonda R, Oliviero F, Scanu A, Campana C, Lorenzin M, Modesti V, Frallonardo P, Punzi L. 2014. FRI0319 Levels of IL-1 and MMP-3 are increased in the synovial fluid from knee osteoarthritis (OA) in patients with concomitant erosive hand OA. Ann Rheum Dis. 71:421.3–422.

[CIT0033] Roman-Blas JA, Jimenez SA. 2006. NF-kappaB as a potential therapeutic target in osteoarthritis and rheumatoid arthritis. Osteoarthr Cartil. 14(9):839–848. 10.1016/j.joca.2006.04.008 16730463

[CIT0034] Sun Y, Liu W, Zhang H, Li H, Liu J, Zhang F, Jiang T, Jiang S. 2017. Curcumin prevents osteoarthritis by inhibiting the activation of inflammasome NLRP3. J Interferon Cytokine Res. 37(10):449–455. 29028430 10.1089/jir.2017.0069

[CIT0035] Taniguchi T, Kurita A, Kobayashi K, Yamamoto K, Inaba H. 2008. Dose- and time-related effects of dexmedetomidine on mortality and inflammatory responses to endotoxin-induced shock in rats. J Anesth. 22(3):221–228. 18685927 10.1007/s00540-008-0611-9

[CIT0036] Toegel S, Wu SQ, Otero M, Goldring MB, Leelapornpisid P, Chiari C, Kolb A, Unger FM, Windhager R, Viernstein H. 2012. *Caesalpinia* sappan extract inhibits IL1β-mediated overexpression of matrix metalloproteinases in human chondrocytes. Genes Nutr. 7(2):307–318. 21850498 10.1007/s12263-011-0244-8 PMC3316743

[CIT0037] Tomita T, Takeuchi E, Tomita N, Morishita R, Kaneko M, Yamamoto K, Nakase T, Seki H, Kato K, Kaneda Y, et al.. 1999. Suppressed severity of collagen-induced arthritis by *in vivo* transfection of nuclear factor kappaB decoy oligodeoxynucleotides as a gene therapy. Arthritis Rheum. 42(12):2532–2542. 10615998 10.1002/1529-0131(199912)42:12<2532::AID-ANR5>3.0.CO;2-2

[CIT0038] Towle CA, Hung HH, Bonassar LJ, Treadwell BV, Mangham DC. 1997. Detection of interleukin-1 in the cartilage of patients with osteoarthritis: a possible autocrine/paracrine role in pathogenesis. Osteoarthritis Cartilage. 5(5):293–300. 9497936 10.1016/s1063-4584(97)80008-8

[CIT0039] Vincenti MP, Brinckerhoff CE. 2001. Early response genes induced in chondrocytes stimulated with the inflammatory cytokine interleukin-1beta. Arthritis Res. 3(6):381–388. 11714393 10.1186/ar331 PMC64850

[CIT0040] Wang C, Zeng L, Zhang T, Liu J, Wang W. 2016. Tenuigenin prevents IL-1β-induced inflammation in human osteoarthritis chondrocytes by suppressing PI3K/AKT/NF-κB signaling pathway. Inflammation. 39(2):807–812. 26846886 10.1007/s10753-016-0309-3

[CIT0041] Xia L. 2010. Histopathology of knee joint synovial tissue and expression of cytokines IL-1β and TNF-α in rabbits with osteoarthritis. Hefei (China): Anhui Medical University.

[CIT0042] Xu B, Zhang WS, Yang JL, Lu N, Deng XM, Xu H, Zhang YQ. 2010. Evidence for suppression of spinal glial activation by dexmedetomidine in a rat model of monoarthritis. Clin Exp Pharmacol Physiol. 37(10):e158–66. 20626414 10.1111/j.1440-1681.2010.05426.x

[CIT0043] Yao H, Chi X, Jin Y, Wang Y, Huang P, Wu S, Xia Z, Cai J. 2015. Dexmedetomidine inhibits TLR4/NF-κB activation and reduces acute kidney injury after orthotopic autologous liver transplantation in rats. Sci Rep. 5:16849. 26585410 10.1038/srep16849 PMC4653646

[CIT0044] Yin D, Zhou S, Xu X, Gao W, Li F, Ma Y, Sun D, Wu Y, Guo Q, Liu H, et al. 2018. Dexmedetomidine attenuated early brain injury in rats with subarachnoid haemorrhage by suppressing the inflammatory response: the TLR4/NF-κB pathway and the NLRP3 inflammasome may be involved in the mechanism. Brain Res. 1698:1–10. 29842860 10.1016/j.brainres.2018.05.040

[CIT0045] Zhang A, Wang S, Zhang J, Wu H. 2016. Genipin alleviates LPS-induced acute lung injury by inhibiting NF-κB and NLRP3 signaling pathways. Int Immunopharmacol. 38:115–119. 27262946 10.1016/j.intimp.2016.05.011

[CIT0046] Zhang C, Boini KM, Xia M, Abais JM, Li X, Liu Q, Li PL. 2012. Activation of Nod-like receptor protein 3 inflammasomes turns on podocyte injury and glomerular sclerosis in hyperhomocysteinemia. Hypertension. 60(1):154–162. 22647887 10.1161/HYPERTENSIONAHA.111.189688 PMC3753400

[CIT0047] Zhang J, Wang Z, Wang Y, Zhou G, Li H. 2015. The effect of dexmedetomidine on inflammatory response of septic rats. BMC Anesthesiol. 15:68. 25929655 10.1186/s12871-015-0042-8 PMC4422264

[CIT0048] Zhang WS, Xu H, Xu B, Sun S, Deng XM, Zhang YQ. 2009. Antihyperalgesic effect of systemic dexmedetomidine and gabapentin in a rat model of monoarthritis. Brain Res. 1264:57–66. 19368840 10.1016/j.brainres.2009.01.029

[CIT0049] Zhong HM, Ding QH, Chen WP, Luo RB. 2013. Vorinostat, a HDAC inhibitor, showed anti-osteoarthritic activities through inhibition of iNOS and MMP expression, p38 and ERK phosphorylation and blocking NF-kappaB nuclear translocation. Int Immunopharmacol. 17(2):329–335. 23856614 10.1016/j.intimp.2013.06.027

[CIT0050] Zhou PH, Liu SQ, Peng H. 2008. The effect of hyaluronic acid on IL-1beta-induced chondrocyte apoptosis in a rat model of osteoarthritis. J Orthop Res. 26(12):1643–1648. 18524010 10.1002/jor.20683

[CIT0051] Zhou R, Yazdi AS, Menu P, Tschopp J. 2011. A role for mitochondria in NLRP3 inflammasome activation. Nature. 469(7329):221–225. 21124315 10.1038/nature09663

[CIT0052] Zhu T, Zhang L, Ling S, Duan J, Qian F, Li Y, Xu JW. 2014. Scropolioside B inhibits IL-1β and cytokines expression through NF-κB and inflammasome NLRP3 pathways. Mediators Inflamm. 2014:819053. 25386048 10.1155/2014/819053 PMC4216717

